# Narratives of hope and concern? Examining the impact of climate scientists’ communication on credibility and engagement

**DOI:** 10.1177/09636625251314159

**Published:** 2025-02-03

**Authors:** Christel W. van Eck, Toni G. L. A. van der Meer

**Affiliations:** University of Amsterdam, The Netherlands

**Keywords:** climate change, climate engagement, climate scientists, credibility, emotions, science communication

## Abstract

Increasingly, more scientists sound the alarm about climate change, sparking debates over the effects of new science communication strategies on scientific credibility. We investigate what happens when climate scientists deviate from science communication that is principally factual and neutral. In an experiment (US sample, *N* *=* 882), we investigated if affective expressions and personal stories impact scientists’ credibility and public climate engagement. The results suggest that when climate scientists incorporate affect or personal anecdotes into their messaging, it does not significantly diminish their credibility. Nevertheless, message consistency is essential; only by aligning the narrative with expressed affect can scientific credibility and climate engagement be increased.

## 1. Introduction

Scientists have for a long time been trained to communicate objectively and neutrally (Douglas, 2009; Head and Harada, 2017), with the idea that communicating scientific facts sufficed to convey their message. Consequently, many scientists have practiced self-censorship when engaging in the climate debate (Boykoff and Oonk, 2018), despite being extremely worried about climate change and the future of the planet (Head and Harada, 2017; Wang et al., 2018). However, times are changing, and with the impacts of climate change becoming progressively more dangerous (IPCC, 2023b), climate scientists feel that they are being ignored. Worldwide, scientists are, for example, increasingly participating in disruptive climate protests, writing open letters to politicians, and organizing themselves in groups like *Scientist Rebellion* (Capstick et al., 2022; Gardner and Wordley, 2019). For instance, in 2022, NASA scientist Peter Kalmus was arrested for chaining himself to the JPMorgan bank in LA, protesting their investment in the fossil-fuel industry. While chained, he gave an emotional speech: “We’ve been trying to warn you guys for so many decades that we’re heading towards a fucking catastrophe, and we’ve been being ignored” (Hanson, 2022).

Scientists continuously navigate a delicate balance between upholding their scientific credibility and raising awareness about the perils of climate change. However, the increasingly severe impacts of climate change compel more and more scientists to reconsider their science communication strategies, to demonstrate their concern. They transition from primarily factual and neutral communication to expressing their genuine feelings about climate change. They argue that this shift is imperative now, as “time is short to secure a livable and sustainable future” (Capstick et al., 2022:773; Gardner and Wordley, 2019).

However, this shift is not embraced by everyone, with critics arguing that it falls outside the purview of a climate scientist’s job description. Traditionally, scientists have been trained to suppress their emotions, as their role was perceived as one of rationality and objectivity (Head and Harada, 2017: 37). Concerns within the academic community have arisen regarding the potential damage to scientists’ credibility, as both the public and fellow academics may perceive it as an abuse of authority and indicative of biased scientific research (Messling, 2020). For decades, the value-free ideal has served as a guiding principle for climate scientists’ communication: “social, ethical, and political values should have no influence over the reasoning of scientists, and that scientists should proceed in their work with as little concern as possible for such values” (Douglas, 2009: 1). For example, the IPCC’s (2023a) motto is to be “policy-relevant,” not “policy-prescriptive.” While many scientists acknowledge the impossibility of achieving pure value-free science (Capstick et al., 2022), a fair share of scientists argue for maintenance of the scientific boundary (Boykoff and Oonk, 2018; Messling, 2020). In this view, scientists should provide objective scientific information to decision-makers while remaining cognizant of the need to uphold scientific integrity (Messling, 2020).

To date, research is still limited in explaining how new forms of science communication impact scientists’ credibility and public climate engagement. Previous research investigated climate scientists’ strategic communication considerations (Boykoff and Oonk, 2018; Post, 2016; Van Eck, 2023) and whether policy-advocacy affects scientists’ credibility (Cologna et al., 2021; Kotcher et al., 2017). However, little attention has been given to the consequences when climate scientists reveal their human side, expressing emotions and personal engagement, potentially compromising their credibility. Therefore, the current study aims to investigate this deviation from strictly objective, factual, and neutral science communication to more affective and personal approaches. More specifically, employing an experimental design, we will test whether climate scientists’ communication of optimistic or pessimistic affect and personal stories influences their scientific credibility and the public’s affective, attitudinal, and behavioral responses to climate change.

## 2. Theoretical framework

### Climate scientists’ communication and credibility

In the field of climate science, researchers encounter numerous challenges when communicating their findings to the public. Like their counterparts in other scientific disciplines, they grapple with common barriers to effective science communication, such as the fear of failure and time constraints. However, climate scientists are also concerned with translating their highly complex science into understandable, engaging, and objective communication (Van Eck, 2023). Previous research indicates that audiences selectively accept climate science information based on its alignment with their cultural worldviews (Kahan et al., 2015), thereby presenting an additional challenge to climate science communication.

Climate scientists feel a responsibility to communicate their findings to policymakers and the public (Cologna et al., 2021; Sharman and Howarth, 2016; Van Eck, 2023), but express concerns about jeopardizing their credibility when engaging in science communication (Boykoff and Oonk, 2018; Van Eck, 2023). While extant research is available on how scientists’ credibility is influenced by their science communication efforts (e.g. Altenmüller et al., 2021; König and Jucks, 2019), less is known about the impacts on climate scientists’ credibility when they incorporate their personal views and feelings on climate change into their public engagement.

Most studies in this area have focused on how advocacy for climate policies affects scientists’ credibility, as well as related concepts such as objectivity, legitimacy, fairness, trust, and honesty (Besley et al., 2021). In our study, we define scientific credibility as “the likelihood of their claims being given weight by non-expert audiences” (Barnes, 2005: 11) and operationalize it empirically as the extent to which the audience perceives the scientist as competent, an expert, intelligent, trustworthy, honest, sincere, caring about society, and sensitive (Kotcher et al., 2017). We view credibility as a multidimensional phenomenon, where various factors contribute to the perception of scientists’ credibility. In the remainder of the article, we avoid using synonyms to ensure consistency in terminology.

Earlier studies have highlighted differing views among scientists on climate advocacy, with some supporting it and others criticizing it (Boykoff and Oonk, 2018; Messling, 2020; Van Eck, 2023). Public perception research shows that the public generally does not condemn scientists for climate advocacy (Cologna et al., 2021; Kotcher et al., 2017). However, nuances exist: Cologna et al. (2021) found that while advocacy doesn’t harm trust, it affects perceived objectivity, and Kotcher et al. (2017) noted that credibility is not reduced unless scientists advocate for nuclear energy. Post and Bienzeisler (2024) observed that scientists’ reputation suffers when policy conclusions are framed as imperatives. Other studies indicate that credibility may be impacted by a perceived high carbon footprint (Attari et al., 2016), but personal or political social media disclosures have little effect (Kim et al., 2024).

### May scientists express their emotions or should they remain neutral?

For decades, the information-deficit model of science communication guided the discussions about climate change. This top–down approach assumes that the public needs “more” or “better” information about climate change and focuses on the “transfer” of knowledge (McDivitt, 2016). However, while this model is still prevalent in research and practice, the field has seen significant development over the past few decades, with increasing emphasis on dialogical approaches (e.g. goals such as “making science/scientists more accessible” and “gaining lay knowledge”) and participatory approaches (e.g. goals such as “participating in research with scientists” and “collectively learning, reflecting, and solving problems”) in science communication (Nerghes et al., 2022: 10). These approaches aim to engage society both intellectually *and* emotionally, which is identified as a promising public engagement strategy (McDivitt, 2016; Nerghes et al., 2022; Weder, 2022). Nonetheless, much remains unknown about how audiences perceive scientists who use emotional communication, particularly given the potential risk to their perceived “neutrality” and “credibility.”

Climate scientists are trained to be scientifically rational, which means they often downplay these emotions to maintain their credibility (Head and Harada, 2017; Willis, 2012). Emotions are “adaptive reactions that are elicited when an event or an object is appraised as relevant to one’s concern” (Brosch, 2021: 16). More broadly, “affect” refers to one’s immediate general feelings about the quality of a stimulus (e.g. good vs bad, positive vs negative) (Cooper and Nisbet, 2016; Slovic et al., 2004; Smith and Leiserowitz, 2014). In contrast, climate “emotions” may include distinct negative emotions such as fear, sadness, and guilt (Brosch, 2021), but also distinct positive emotions like optimism, pride, and gratitude (Schneider et al., 2021). Numerous public accounts exist about how climate scientists experience intense negative emotions about the reality and severity of climate change through their work (e.g. Willis, 2012, for a scientific account). One study showed that hope, anger, and frustration were emotions most often expressed by climate scientists (Wang et al., 2018). Another study identified how climate scientists manage their painful emotions. Their most profound strategy was “blocking or not acknowledging emotional responses to confronting data about the consequences of inaction on climate change” for the reason that “dispassion and rationality [are] understood as central to the role of the scientists in producing reliable data” (Head and Harada, 2017: 37).

While scientists are often hesitant to express their emotions, there are reasons to assume that communicating one’s emotions can be beneficial for fostering climate engagement. Previous research measured climate engagement at various levels, encompassing people’s affective, attitudinal, and behavioral responses to climate change. These studies suggest that all of these responses are distinct constructs, interrelated in complex, nonlinear ways, and leading to diverse outcomes (Chapman et al., 2017; Xie et al., 2019). We define climate engagement as the public’s affective responses, their risk perceptions of climate change (i.e. attitudinal responses), and their behavioral willingness to take climate action (i.e. behavioral responses).

A significant body of research showed that affect is among the most influential predictors of variations in people’s climate change risk perceptions, surpassing factors such as their knowledge about climate change (Van der Linden, 2015; Van Eck et al., 2020; Xie et al., 2019). Although the precise relationship between emotions and desired outcomes is intricate (Chapman et al., 2017), emotions typically have a positive effect on support for climate polices (Leiserowitz, 2006; Smith and Leiserowitz, 2014; Wang et al., 2018). In particular, optimistic climate messages are promoted as effective persuasive strategy for climate engagement (Morris et al., 2020). Fear appeals can for example lead to unintended consequences, causing people to distance themselves from the issue (O’Neill and Nicholson-Cole, 2009). Similarly, aggressive communication styles in science blogs about climate change could evoke psychological reactance and violate expectations (Yuan and Lu, 2020). However, Morris et al. (2020) put this assumption to the test, and found that pessimistic affective messaging increased all levels of climate engagement. Therefore, if climate scientists communicate an affective pessimistic view of climate change, it may elicit strong affective responses, while leaving people’s attitudes and behaviors unchanged.

We will focus on affect (pessimistic or optimistic) rather than specific emotions because scientists’ communication is often characterized by an overall affective tone rather than a single distinct emotion. Problematically, previous research has not actually tested whether it is legitimate that scientists fear losing their credibility when expressing their affective evaluations of climate change. It also has not explored whether climate scientists communicating their affective responses has a positive effect on climate engagement. Therefore, we pose the following research question:

RQ1: How does scientists’ communication of a neutral versus an affective (pessimistic or optimistic) view of climate change affect scientists’ credibility and people’s affective, attitudinal, and behavioral responses to climate change?

### May scientists share personal stories or should they stick to the facts?

Climate scientists’ strong commitment to objectivity (Head and Harada, 2017) often leads to communication that is primarily factual. However, scientists are increasingly being trained to utilize storytelling as a promising method for effectively conveying their research. Personal stories are subjective accounts of one’s experiences in the world (Maynes et al., 2008). The subjectivity in personal stories is at odds with objectivity in factual accounts. While the latter is historically part of the comfort zone of scientists, it is inevitable that scientists also have personal stories about one’s experiences with climate change; however, scientists might be afraid to share such stories publicly in an attempt to remain objective.

However, personal stories are generally promoted as a promising climate change communication strategy (Gustafson et al., 2020; Moezzi et al., 2017), also for scientists (Corner and Van Eck, 2014). Gustafson et al. (2020) explains:

Because people tend to view climate change as distant and abstract, stories that translate information about the effects of climate change into relatable and concrete personal experiences may be especially effective at reducing psychological distance and increasing emotional engagement, thereby increasing perceived importance and risk perceptions. (p. 122)

We define both personal stories and factual accounts as forms of narratives, where the higher the narrative structure, the more story-like the narrative and less factual (Morris et al., 2019). A high narrative structure—“with a beginning, middle, and end—ties actions and implications together in a causal chain, rather than relying on a set of propositions that may be more or less well integrated” (Green, 2006: 164). High narrative structures often present the lived experience of a character driving the action and experiencing atypical events (Cooper and Nisbet, 2016). Increasingly more studies provide evidence that personal stories have a positive impact on climate engagement (Gustafson et al., 2020; Morris et al., 2019), but exceptions exist (Jones, 2014). For example, Morris et al. (2019) showed how narratives structured as personal stories elicited more climate engagement than narratives with factual information.

However, it remains unknown whether scientists’ fear of damaging their credibility by deviating from the facts is justified. By extension, it is also unknown whether climate scientists sharing their personal stories about climate change promote climate engagement. Hence, our second research question is as follows:

RQ2: How does scientists’ use of a personal story versus a factual account about climate change affect their credibility and people’s affective, attitudinal, and behavioral responses to climate change?

In addition, previous research has shown that personal stories about climate change have a stronger impact when they evoke emotions (Gustafson et al., 2020; Morris et al., 2019). Consequently, we expect that personal stories with affective expressions likely elicit stronger climate engagement. In contrast, factual accounts without affective expressions may align more with traditional perceptions of scientists, thereby increasing their credibility. Therefore, we are interested in exploring this interaction as follows:

RQ3: How do scientists’ narrative structure, in conjunction with their affective expressions, affect their credibility and people’s affective, attitudinal, and behavioral responses to climate change?

### Moderation

Earlier research has shown that the public’s trust in scientists is not significantly impacted when scientists engage in climate advocacy (Cologna et al., 2021; Kotcher et al., 2017). However, extant research also demonstrates how people’s distrust in the climate scientific community is associated with climate skepticism (Sarathchandra et al., 2021; Van Eck et al., 2020). Thus, understanding the role of people’s trust in the climate scientific community could potentially explain divergent results regarding scientists credibility and public climate engagement, as follows.

RQ4a: Does people’s trust in the climate scientific community moderate the relationship between exposure to different message types (affective expression and narrative structure) and the perceived credibility of the scientist as well as public climate engagement?

Furthermore, previous research has indicated that people’s personal experiences with extreme weather events predict the extent to which they perceive climate change as a risk (Van der Linden, 2015; Van Eck et al., 2020; Xie et al., 2019). Extreme weather events are also identified as an important gateway for communicating about climate change (Marshall, 2014). Therefore, the relationship between how the audience perceives narratives about extreme weather events might be influenced by their own experiences with such events, as follows:

RQ4b: Do people’s experiences with extreme weather events moderate the relationship between exposure to different message types (affective expression and narrative structure) and the perceived credibility of the scientist as well as public climate engagement?

Finally, while increasingly more is known about the impact of optimistic and pessimistic climate change messaging (e.g. Morris et al., 2020), there is less understanding of how the personal traits of the receiver moderate this relationship, such as their own level of optimism. One study suggests that optimists tend to spend less time on climate change arguments supported by a scientific consensus (Beattie et al., 2017), which could affect how they perceive emotionally intense messages from climate scientists. We expect that pessimistic individuals are more sensitive to messages from climate scientists, and that therefore individuals’ optimistic traits moderate their level of climate engagement. We propose the following research question:

RQ4c: Does individuals’ optimism moderate the relationship between exposure to different message types (affective expression and narrative structure) and the perceived credibility of the scientist as well as public climate engagement?

## 3. Methodology

We conducted an online randomized experiment among a sample of the general public of the United States (US) to understand how a scientist’s messaging strategy impacts their credibility and the respondents’ engagement with climate change.

### Research design and stimuli

The project is preregistered on AsPredicted^1^ (https://aspredicted.org/8h34-htfd.pdf) and the LAB ethics review board of the University of Amsterdam granted ethical approval (2022-CC-15339). We employed a 3 (scientists’ affective expression: pessimistic vs optimistic vs neutral) × 2 (narrative structure: personal story vs factual account) between-subjects factorial design. We rely on Kotcher et al. (2017), who designed for their study on climate advocacy by scientists’ stimuli in which Professor Wilson writes Facebook posts about an interview that he gave for the Associated Press. To maximize the authenticity of their stimuli, they included a fake hyperlink to the interview at the end of the post. We followed this exact same approach, only the Facebook posts were replaced with blog posts (see Supplement). The design of the blog post was consistent across all conditions. For ecological validity, the content of the blog posts was inspired by the themes identified in Wang et al.’s (2018) study, in which they asked climate scientists to write a letter about how they emotionally experience climate change. Prominent themes included “hope,” “the world/planet/Earth,” “future generations,” “humanity”, “close relationships,” and “climate consequences.”

In the affective conditions, we manipulated sentences in the blog posts in which Professor Wilson communicated his emotions about the future of the planet. The content of the blog post that respondents saw, highlighted that Professor Wilson was either optimistic or pessimistic about the future of the planet, or not communicating any emotions about the future of the planet. Only the sentences with emotional expressions were manipulated and no other information was added. The optimistic condition included statements like “I am confident that we can mitigate the dangerous impacts of climate change to ensure the future of my children,” opposed to the pessimistic condition with statements like “I am extremely scared, as I foresee that it will be immensely challenging for society to implement the systemic change that is needed.” The neutral condition avoided affective language and contained statements like “the question is whether we can mitigate the dangerous impacts of climate change.”

In the narrative structure conditions, we manipulated one paragraph in which Professor Wilson communicated either purely factual information or shared a personal story, without adding new information. Respondents were either exposed to blog posts in which Professor Wilson shared a personal story about how he and his family experienced a heat wave in his hometown. Statements like “I was planning to bring my kids to school when I received a phone call from their teacher that the school would close that day, due to sweltering temperatures in the classrooms” were included in these stimuli. This high narrative structure included a lived experience (e.g. “drops of sweat slid off all of our heads”) of a main character (Professor Wilson) experiencing an atypical event (schools closing due to high temperatures). Contrastingly, in the factual accounts (i.e. low narrative structure), Professor Wilson shared facts about heat waves in the US, relying on data of the US Environmental Protection Agency (https://www.epa.gov/climate-indicators/climate-change-indicators-heat-waves). For example, these stimuli included the following statement: “Heat waves are occurring three times more often than they did in the 1960s—about six per year compared with two per year.”

The survey flow was designed as follows. First, respondents signed an informed consent form. Subsequently, they filled out questions for the moderator and control variables. After an attention check and a statement to read the following page carefully, respondents were exposed to one of the stimuli for at least 20 seconds. Subsequently, respondents’ emotional arousal was measured immediately with a question. Then, questions for the manipulation checks and dependent variables followed. The survey ended with a debrief, in which respondents were informed about the main questions of the study, the importance, and how to know more about climate change and to follow-up with the researchers.

### Sample

We focused on the US for two main reasons: first, the country has seen a shift in communication strategies among individual climate scientists, who are increasingly vocal in public debates (e.g. early example James Hansen, recent example Peter Kalmus). This shift is occurring alongside ongoing debate among US climate researchers about the appropriate role of scientists in climate advocacy. Second, the US public exhibits deep polarization on climate change (Bolsen and Shapiro, 2018), reflecting how the perception of climate science communication in this country is more strongly driven by cultural worldviews than by a technical understanding of the facts (Kahan et al., 2015).

Data were collected with Kantar Lightspeed, a large international research agency that relies on a large database of respondents from different cultures. We collected 918 responses from the US general public. We set quotas on gender, age, and education to match census data provided by the panel company, aiming to approximate representativeness (see Supplement). After cleaning the dataset by removing speeders, straightliners, and respondents that did not finish the survey, our final sample size was 882. Respondents were randomly assigned to the six conditions where 446 of the respondents saw the personal story and 436 the factual account. Furthermore, 303 respondents were exposed to the optimistic condition, 294 to the pessimistic condition, and 285 to the neutral condition. Post hoc randomization checks using ANOVA and chi-squared tests confirmed that there were no significant differences in age, gender, political affiliation, or education level across the six experimental conditions, indicating successful randomization.

On average, respondents were 46.72 years old (*SD* = 17.65) and 46.8% of them identified as female. Furthermore, 34% of the respondents identified as Republican or leaned toward the right-wing, 19.8% as Independent, and 37.5% as Democrat or leaned toward the left-wing. Finally, 40.1% of the respondents had not finished high school or only had a high school diploma or equivalent, 16.7% had completed technical or vocational school, some college without a degree, or had an associate’s or 2-year college degree, and 43.2% had attained a 4-year college degree or higher, including graduate or professional degrees and PhDs.

### Measures

*Scientists’ credibility* was measured with an 8-item 7-point Likert-type scale of Kotcher et al. (2017), derived from McCroskey and Teven (2009). Respondents assessed to what extent they agreed with statements about Professor Wilson, for example, they rated the extent to which they found him an expert and trustworthy (*M* = 4.97; *SD* = 1.23; Cronbach’s alpha = .94).^2^

*Affective, attitudinal, and behavioral responses to climate change* were measured with three scales. Respondents’ emotional arousal was measured with a 1-item Likert-type scale, which asked whether they agreed the blog post emotionally intense to read, rated from 1 (*strongly disagree*) to 7 (*strongly agree*) (*M* = 4.13; *SD* = 1.74).^3^ With eight 7-point items, the climate change risk perception scale of Van der Linden (2015) measured to what extent the respondents perceive climate change as a risk (e.g. “ I am concerned about climate change”) (*M* = 4.81; *SD* = 1.57; Cronbach’s alpha = .96). Finally, respondents’ personal behavioral willingness to take climate action was measured with six 7-point items of Xie et al. (2019) (e.g. “After reading the article, I would personally be willing to pay more for and use less electricity”) (*M* = 4.06; *SD* = 1.60; Cronbach’s alpha = .93).

#### Moderation

Respondents’ trust in the climate scientific community was measured with a 1-item Likert-type scale (adapted from Hmielowski et al., 2014) (*M* = 2.87; *SD* = 1.61). Furthermore, respondents were asked how often they personally experienced any type of extreme weather event in their local area (never, once, twice, three times, or more; Van der Linden, 2015; *M* = 3.27; *SD* = 1.56). Finally, with a 5-item 7-point Likert-type scale, respondents answered statements about the extent to which they regard themselves as optimistic (*M* = 3.88; *SD* = 1.52; Cronbach’s alpha = .90) (adapted scale of Life Orientation Test; Scheier and Carver, 1985). All measures were used as separate moderators.

### Manipulation checks

The stimuli were first pilot tested on MTurk with a smaller sample to verify whether the stimuli were manipulated successfully. Both experimental conditions were successfully manipulated in the second round of testing. The manipulations were also successful in the final dataset (see Supplement).

## 4. Results

### Affective expressions

To answer RQ1, which focused on the effect of emotional communication, a univariate analysis was performed to compare how Professor Wilson’s expression of either a pessimistic, optimistic, or neutral view on climate change affected his credibility. This test revealed that there was no statistically significant difference at the *p* < .05 level in mean credibility scores between the pessimistic (*M* *=* 5.01, *SD* *=* 1.16), optimistic (*M* *=* 5.02, *SD* *=* 1.24), and neutral (*M* *=* 4.86, *SD* *=* 1.28) groups (*F*(2, 876) = [1.447], *p* = .24). Thus, whether Professor Wilson communicates optimistically, pessimistically, or remains neutral did not result in a difference in his perceived credibility.

In addition, three separate univariate analyses were conducted to compare how Professor Wilson’s expression of either a pessimistic, optimistic, or neutral view on climate change affected people’s emotional responses, risk perceptions, and behavioral willingness to take climate action. At the *p* < .05 level, these tests revealed that there was no statistically significant difference in mean risk perception scores between the pessimistic (*M* *=* 4.86, *SD* *=* 1.53), optimistic (*M* *=* 4.8, *SD* *=* 1.62), and neutral (*M* *=* 4.76, *SD* *=* 1.55) groups (*F*(2, 879) = [0.304], *p* = .74).

Likewise, there was no significant difference for mean behavioral willingness scores between the pessimistic (*M* *=* 4.1, *SD* *=* 1.6), optimistic (*M* *=* 3.99, *SD* *=* 1.59), and neutral (*M* *=* 4.11, *SD* *=* 1.62) groups (*F*(2, 879) = [0.515], *p* = .6). Thus, if Professor Wilson varies in communicating either optimistically, pessimistically, or neutrally one emotional expression does not elicit stronger attitudinal or behavioral responses than the others.

However, at the *p* < .05 level, there was a significant effect on people’s emotional responses (*F*(2, 879) = [13.158], *p* < .01, ηp^2^ = .029). A post hoc comparison using a Bonferroni test indicated that the mean score for the pessimistic condition (*M* = 4.55, *SD* = 1.69) was significantly higher than the neutral condition (*M* = 3.87, *SD* = 1.74; *M_diff_* *=* 0.68, *p* < .01) and optimistic condition (*M* = 3.98, *SD* = 1.74; *M_diff_* *=* 0.57, *p* < .01). The difference between the optimistic and neutral condition was not significant. Thus, if Professor Wilson affectively expresses himself pessimistically, it elicits stronger affective responses by the public than expressing himself optimistically or neutrally.

Overall, to answer RQ1, across all the conditions, Professor Wilson’s credibility was relatively high. These results suggest that if scientists either communicate an optimistic or pessimistic view on climate change, their affective expressions do not compromise their scientific credibility more than remaining neutral. The results also indicate that such affective expressions do not impact the public’s attitudinal and behavioral responses to climate change more than remaining neutral, except that scientists potentially elicit stronger affective responses when they communicate pessimistically.

### Narrative structure

To answer RQ2, likewise, a univariate analysis was conducted to compare how Professor Wilson’s use of a personal story or factual narrative impacts his scientific credibility. This test showed that there was no statistically significant difference at the *p* < .05 level in mean credibility scores between the factual account (*M* *=* 4.97, *SD* *=* 1.27) and personal story (*M* *=* 4.97, *SD* *=* 1.19) groups (*F*(1, 876) = [0.001], *p* = .97). Thus, if Professor Wilson either communicates a personal story or factual account, his chosen narrative structure does not change his credibility.

Furthermore, three separate univariate analyses pointed out that the effects of Professor Wilson’s narrative structure use did not significantly impact people’s emotional arousal between the factual account (*M* *=* 4.07, *SD* *=* 1.8) and personal story (*M* *=* 4.2, *SD* *=* 1.7) groups (*F*(1, 880) = [1.241], *p* = .27). Similarly, the mean risk perception scores between the factual account (*M* *=* 4.78, *SD* *=* 1.59) and personal story (*M* *=* 4.83, *SD* *=* 1.54) groups were not significant (*F*(1, 880) = [0.242], *p* = .62). Finally, there were also no significant mean behavioral willingness score differences across the factual account (*M* *=* 4, *SD* *=* 1.59) and personal story (*M* *=* 4.13, *SD* *=* 1.61) groups (*F*(1, 880) = [1.533], *p* = .22). Hence, either communicating a factual account or personal story does not elicit stronger affective, attitudinal, or behavioral responses to climate change.

Generally, to answer RQ2, Professor Wilson’s credibility was relatively high in both conditions. These results suggest that if scientists either use a personal story or factual account, one narrative structure does not compromise their scientific credibility more than the other. The results also indicate that communicating a personal story does not have a stronger impact on the public’s affective, attitudinal, or behavioral responses to climate change than communicating a factual account.

### Interaction effects: Emotional expressions × narrative structure

**Figure 1.**
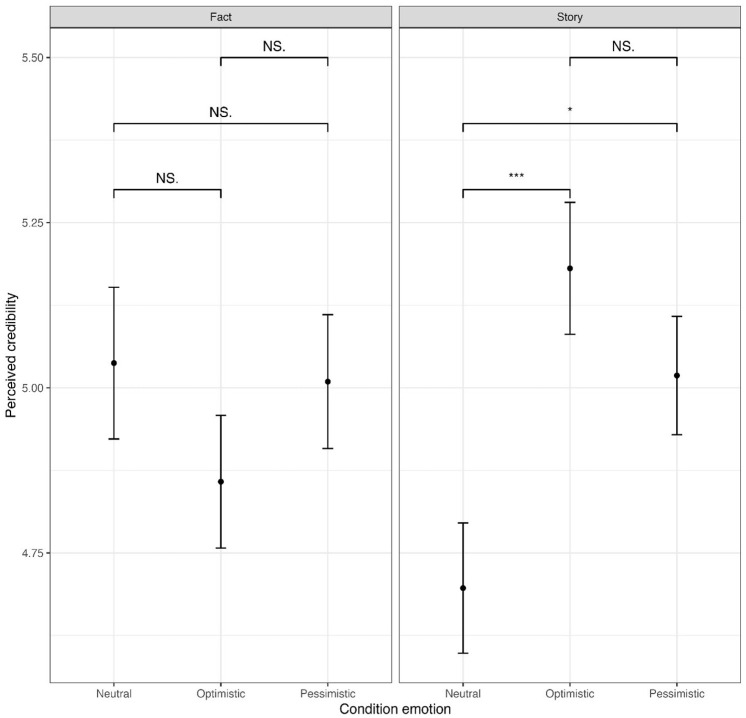
Mean scores perceived credibility. Asterisks indicate significance levels (*p < .05; ***p < .001), and NS = not significant.

RQ3 was formulated to test the combination between both experimental factors. At the *p* < .05 level, a significant result was found for interaction effects of the experimental conditions, affective expressions, and the narrative structure on Professor Wilson’s scientific credibility (*F*(2, 876) = [5.416], *p* = .005, ηp^2^ = .012) (Figure 1). Post hoc comparisons using a Bonferroni test indicated that if Professor Wilson communicates a personal story that the mean score for the optimistic condition (*M* = 5.18, *SD* = 1.22) was significantly higher than the neutral condition (*M* = 4.7, *SD* = 1.2; *M_diff_* *=* 0.48, *p* < .01). No significant differences were found between the pessimistic condition and either the optimistic or neutral condition. Likewise, no significant differences were found when comparing the emotional conditions for when Professor Wilson communicates a factual account.

**Figure 2.**
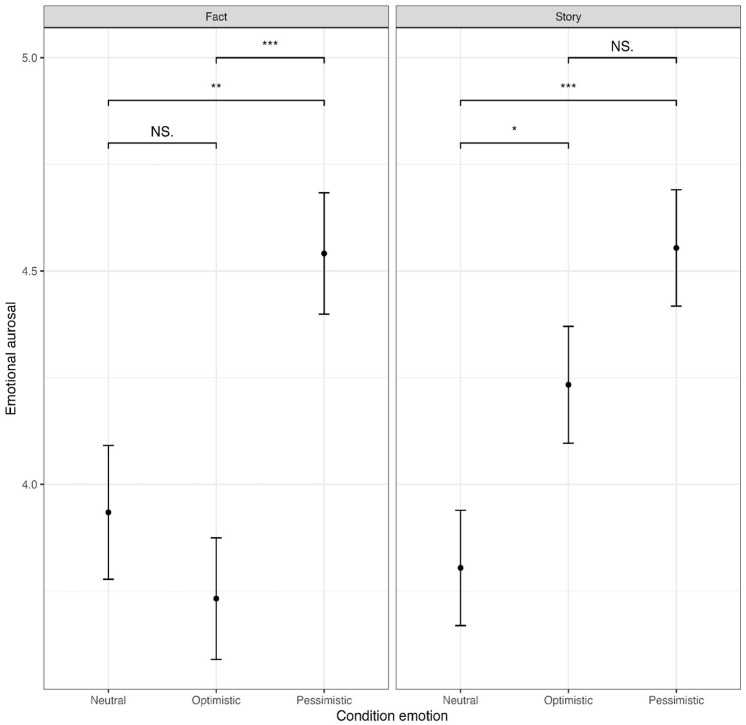
Mean scores emotional arousal. Asterisks indicate significance levels (*p < .05; **p < .01; ***p < .001), and NS = not significant.

**Figure 3.**
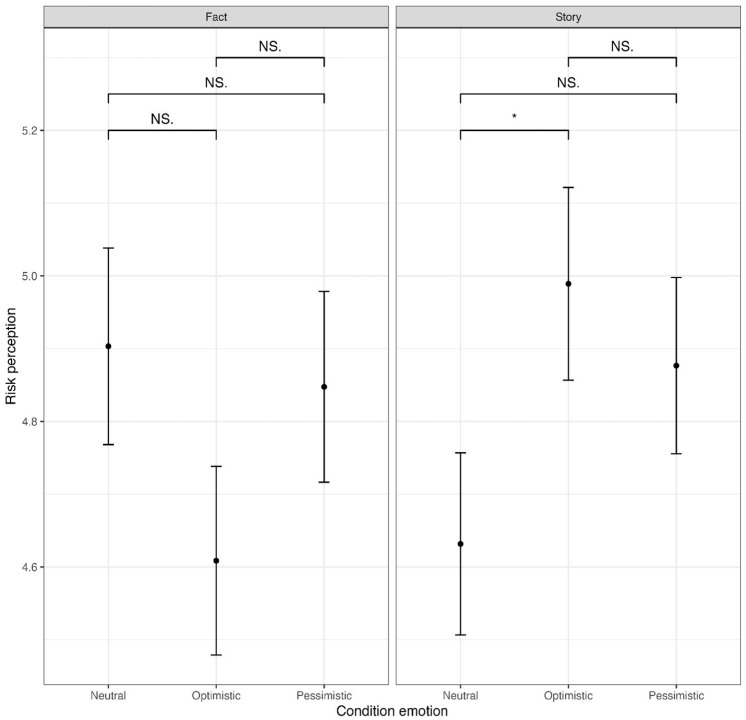
Mean scores risk perception. Asterisk indicates significance levels (*p < .05), and NS = not significant.

**Figure 4.**
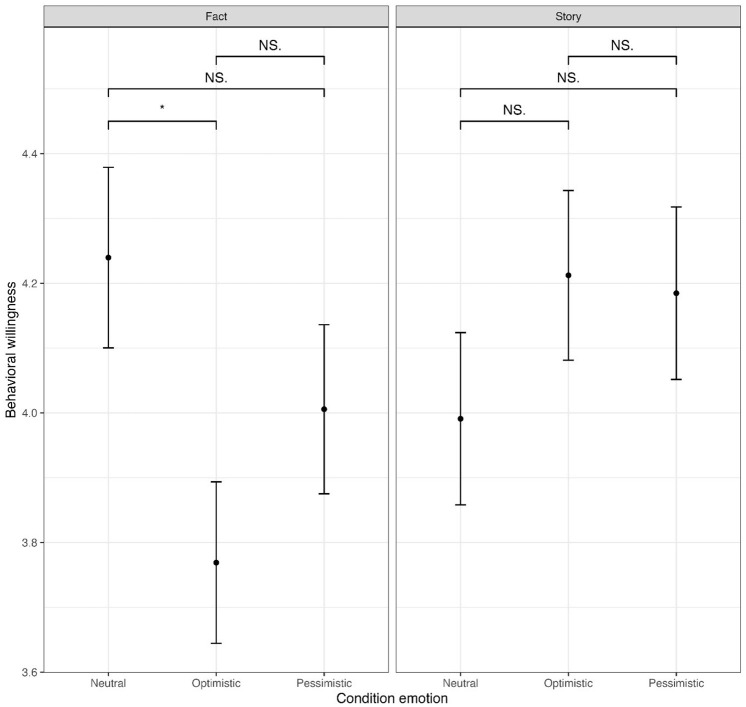
Mean scores behavioral willingness. Asterisks indicate significance levels (*p < .05; **p < .01; ***p < .001), and NS = not significant.

Furthermore, at the *p* < .05 level, significant results were also found for interaction effects of the experimental conditions affective expressions and the narrative structure on the public’s attitudinal and behavioral responses (Figures 2 to 4). A significant effect was found for people’s risk perceptions (*F*(2, 876) = [3.197], *p* < .05, ηp^2^ = .007). However, a post hoc Bonferroni test indicated no significant results, which could be attributed to the Bonferroni correction. Another significant effect was found for people’s behavioral willingness to take climate action (*F*(2, 876) = [3.490], *p* < .05, ηp^2^ = .008). The Bonferroni test revealed that if Professor Wilson communicates a factual account that the mean score of the neutral condition (*M* = 4.24, *SD* = 1.63) was significantly higher compared with the optimistic condition (*M* = 3.77, *SD* = 1.54; *M_diff_* *=* 0.47, *p* < .05).

Overall, to answer RQ3, these interaction effects indicate that if scientists use a personal story as narrative structure, their scientific credibility is higher when they use affective expressions. In contrast, if they use a factual account as narrative structure, their scientific credibility is higher when they remain neutral. Moreover, the results suggest that if scientists use a factual account as narrative structure, they elicit stronger behavioral responses when they remain neutral, while for eliciting affective and attitudinal public responses the narrative structure does not matter.

### Moderation effects

For the final RQ4, we asked how people’s (a) trust in the climate scientific community, (b) personal experiences with extreme weather events, and (c) their optimistic trait moderates the relationships studied in RQ1 and RQ2. For this purpose, we ran several ordinary least squares (OLS) regression models, using the four outcome variables—that is, perceived credibility, emotional arousal, risk perception, behavioral willingness—as dependent variables and the different conditions in combination with one of the three moderators as predicting variables. Table 1 reports the moderating effects with personal experience, Table 2 with trust, and Table 3 with optimistic trait (see Supplement).

Inspecting the three different tables highlights that it is, comparatively speaking, primarily people’s trust in the climate scientific community that plays a role in the effect of how climate scientists communicate. For example, among those with higher trust in the community, perceived credibility (Table 2, Model 1) is higher when exposed to a personal story with neutral (*p* < .05) or optimistic (*p* < 0.1) perspective rather than the more neutral and factual approach. Similarly, behavioral willingness is higher among those with higher trust in the condition where respondents were exposed to a personal story with an optimistic tone (*p* < 0.1) (Table 2, Model 4). Risk perceptions were found to be lower among those scoring high on trust when exposed to a personal story with a pessimistic tone (*p* < 0.1) (Table 2, Model 3). The relationship between the different conditions and respondents’ emotional arousal was found to be unaffected by people’s prior trust in the climate scientific community (Table 2, Model 2). In sum, those who have more trust in the community are more open to personal stories of climate scientists, as long as these narratives remain optimistic or neutral.

From Table 1, it can be concluded that for the moderating role of personal experiences with extreme weather events there was less significant interactions. Only for the dependent variable, behavioral willingness, was the moderating role of personal experiences with extreme weather events was significant (*p* < .05) (Table 1, Model 4). Those respondents who scored higher on personal experience would score lower on behavioral willingness after being exposed to a personal story that is neutral compared with a factual message with a natural tone.

Table 3 shows that respondents’ optimistic trait did not moderate the effect between the experimental conditions and the dependent variables.

## 5. Discussion

An increasing number of scientists sound the alarm about dangerous climate change (Capstick et al., 2022; Gardner and Wordley, 2019). However, some express concerns that such practices may hurt scientists’ credibility. Our study aimed to further explore the implications when scientists depart from science communication that is principally factual and neutral and engage in emotional and personalized science communication. In summary, our findings suggest that climate scientists can uphold their credibility while communicating their affective optimism or pessimism, and personal stories. However, maintaining message consistency is crucial; scientific credibility and climate engagement can only be enhanced when the narrative is aligned with the emotional expressions.

We demonstrate that scientists’ credibility is not necessarily compromised when they communicate their affective optimism or pessimism (*RQ1*) or share personal stories (*RQ2*). Therefore, climate scientists could consider communicating their genuine emotions instead of intentionally suppressing them in an attempt to appear rational to the public (Head and Harada, 2017). This approach may be particular promising for audiences with high trust in the climate scientific community, especially when sharing personal stories with a neutral or optimistic perspective (*RQ4)*. This result is consistent with more recent literature that emphasizes bringing science to life and engaging audiences emotionally (Corner and Van Eck, 2014; Weder, 2022). Overall, this finding complements earlier research on climate advocacy (Cologna et al., 2021; Kotcher et al., 2017) and provides insight from the public’s perspective into the ongoing debate about whether scientists’ credibility is undermined when scientists incorporate their own views into their communication. However, the existing evidence on this topic is still limited, and further extensive research is necessary to fully understand how alternative science communication strategies affect climate scientists’ credibility.

The evidence should be interpreted with caution, as our results also suggest that scientists need to be consistent in their messaging. The study showed that generally scientists’ credibility and public responses to climate change are highest when the narrative structure aligns with their affect (*RQ3*). If a climate scientist communicates a factual account, it is most effective if the scientist remains more neutral in their expressions, whereas if this scientist communicates a personal story, it is most effective to express optimistic or pessimistic affect. This finding nuances previous literature that advocates for increased use of storytelling in science (e.g. Corner and Van Eck, 2014) and climate change communication (e.g. Gustafson et al., 2020) by demonstrating that the effectiveness of personal stories is context-dependent (i.e. messenger, message, audience) and therefore only effective under certain circumstances. Drawing from existing research on expectancy violation theory (Afifi and Metts, 1998; Yuan et al., 2019; Yuan and Lu, 2020) and scientists’ role conceptions (Messling, 2020), potentially, society primarily favors consistency in scientists, expecting that scientists either step into the role of a “factual, objective, and neutral scientist” or into the role of a “concerned citizen.” This finding builds upon Yuan et al.’s (2019) study, suggesting that a polite communication strategy violates the public’s expectations, arguably making scientists “look weak and vacillating” (p. 285) compared with neutral and aggressive styles. Future research should point out whether expectation violation theory indeed provides an explanation for this finding, for example, in the context where scientists participate in civil disobedience and disruptively violate the public’s expectations. Furthermore, more research is needed on *how* the public differentiates between the two roles.

Contrary to the findings of Morris et al. (2020), our results indicate that when a scientist communicates pessimistically, it elicits stronger emotional responses among the public but does not lead to increased risk perceptions or a great willingness to take climate action (*RQ1*). This outcome may be explained by the notion that attitudinal and behavioral responses are more resistant to change, as they are influenced by a wide range of cognitive, experiential, and socio-cultural influences (Van der Linden, 2015; Van Eck et al., 2020; Xie et al., 2019). This finding underscores once again the intricate relationship between emotions and desired outcomes (Chapman et al., 2017). Furthermore, while the data suggest that in certain circumstances, scientists sharing personal stories can have a positive impact on climate engagement (Gustafson et al., 2020; Moezzi et al., 2017), it is not necessarily a universally effective climate change communication strategy for scientists (Jones, 2014). The success of this approach also depends on the audience, as the results suggest that sharing affective personal stories is less effective for audiences with lower trust in the climate scientific community (*RQ4a*).

As with any study, one should bear in mind the limitations. First, our research focused on the US public, but scientists are potentially more afraid of their colleagues judging their actions than the public (Van Eck, 2023). Therefore, it is recommended to replicate this study among US scientists. Second, we investigated Professor Wilson’s credibility, which means that we cannot make any inferences about the impact on the scientific community as a whole or the climate science discipline. Third, due to the constraints of a forced-exposure experiment, we used a blog post, which might fit better than other formats with the information-deficit paradigm. Future research should compare this format with scientific publications or interactive formats like workshops and art-based interventions, which align with dialogical and participatory models of science communication (Nerghes et al., 2022; Weder, 2022). Fourth, the personal story stimuli could have included a longer and more complex narrative, allowing for stronger narrative transportation and identification (Cooper and Nisbet, 2016; Green, 2006), which should be considered for future research. Fifth, although our stimulus was designed to be gender-neutral, the gendered perception of scientists and the expression of emotions in American society may have influenced respondents. Future research should consider replicating this study with explicit male or female representations of Professor Wilson to explore potential gender biases in greater depth. Last, we used “scientific credibility” as a measure. However, it would be interesting to see whether our results uphold if equivalent measures were used such as “objectivity” and “trustworthiness” (Besley et al., 2021).

In conclusion, our study provides valuable insights into how climate scientists can balance emotional expression with factual integrity to drive meaningful action on climate change while maintaining their credibility.

## Supplemental Material

sj-docx-1-pus-10.1177_09636625251314159 – Supplemental material for Narratives of hope and concern? Examining the impact of climate scientists’ communication on credibility and engagement Supplemental material, sj-docx-1-pus-10.1177_09636625251314159 for Narratives of hope and concern? Examining the impact of climate scientists’ communication on credibility and engagement by Christel W. van Eck and Toni G. L. A. van der Meer in Public Understanding of Science

## Notes

1. We had preregistered our focus on specific emotions. However, since the manipulation checks for these emotions failed, we shifted our focus to the overall affective tone (optimistic or pessimistic) (see Supplement).

2. We preregistered that scientists’ credibility would also be measured with two 1-item scales about Professor Wilson’s perceived goal to inform and persuade. However, we ultimately decided to exclude these items due to space constraints, as they did not substantially alter the overall narrative.

3. We preregistered that emotional arousal would be measured with two items. Yet, we decided to exclude one item about the extent to which respondents felt optimistic, neutral, or pessimistic after reading the blog post, since the reliability score of the two-item measure was dissatisfactory (Cronbach’s alpha = .34).
