# Supplementary material for: Narratives of hope and concern? Examining the impact of climate scientists’ communication on credibility and engagement
Source: Public Underst Sci. 2025 Feb 3;34(6):734–51. doi: 10.1177/09636625251314159 (PMC12274565; doi:10.1177/09636625251314159)
Supplement: sj-docx-1-pus-10.1177_09636625251314159 – Supplemental material for Narratives of hope and concern? Examining the impact of climate scientists’ communication on credibility and engagement [file sj-docx-1-pus-10.1177_09636625251314159.docx]

**Narratives of Hope and Concern?**

***Examining the Impact of Climate Scientists’ Communication on Credibility and Engagement***

Christel W. van Eck & Toni van der Meer

**Supplemental Materials**

1. Stimuli (p.2)
2. Soft quotas (p.8)
3. Manipulation checks (p.9)
4. Tables with results (p.10)

**Stimuli**

**Pessimistic x personal story**


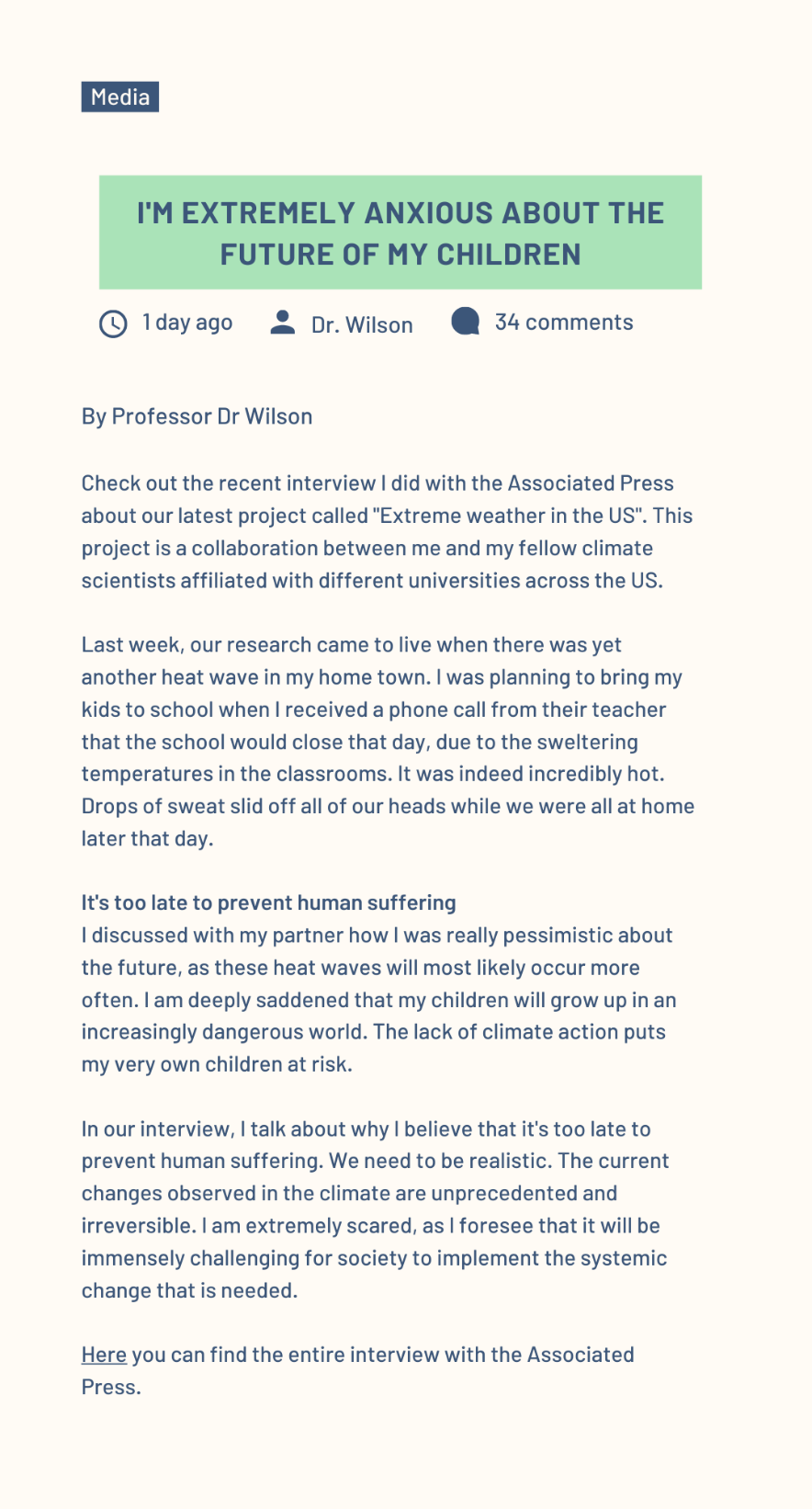


**Pessimistic x factual account**


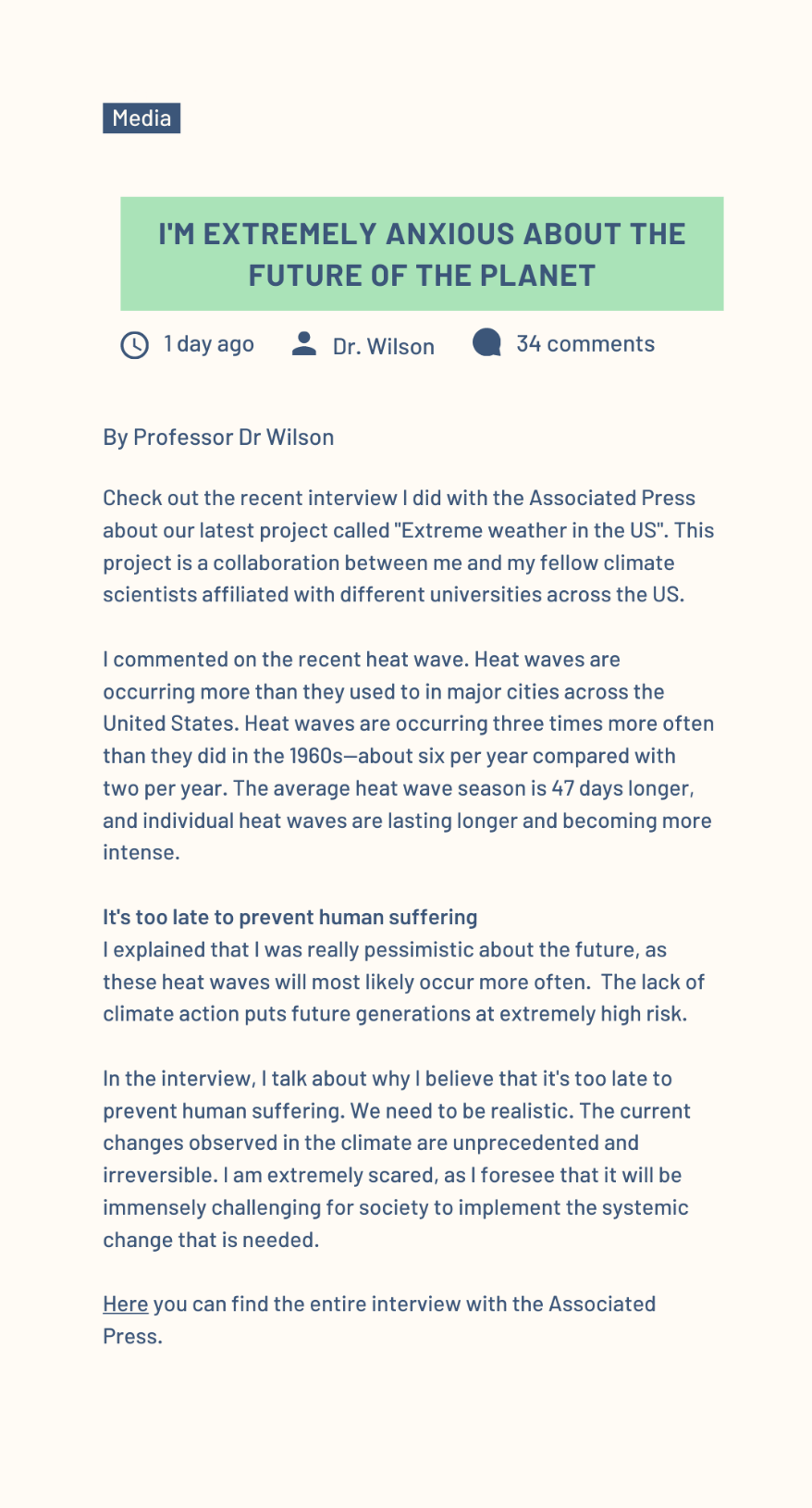


**Optimistic x personal story**


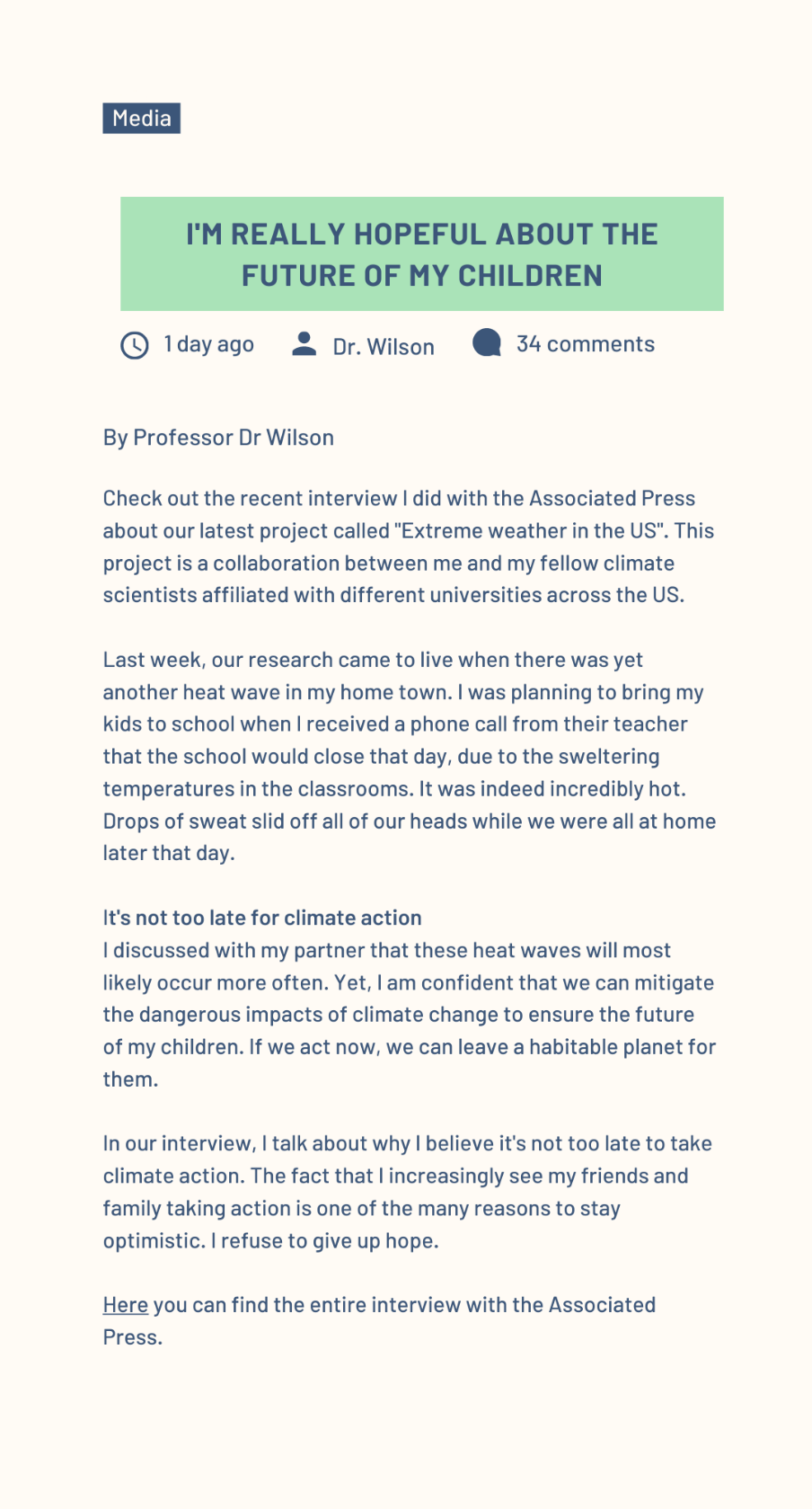


**Optimistic x factual account**


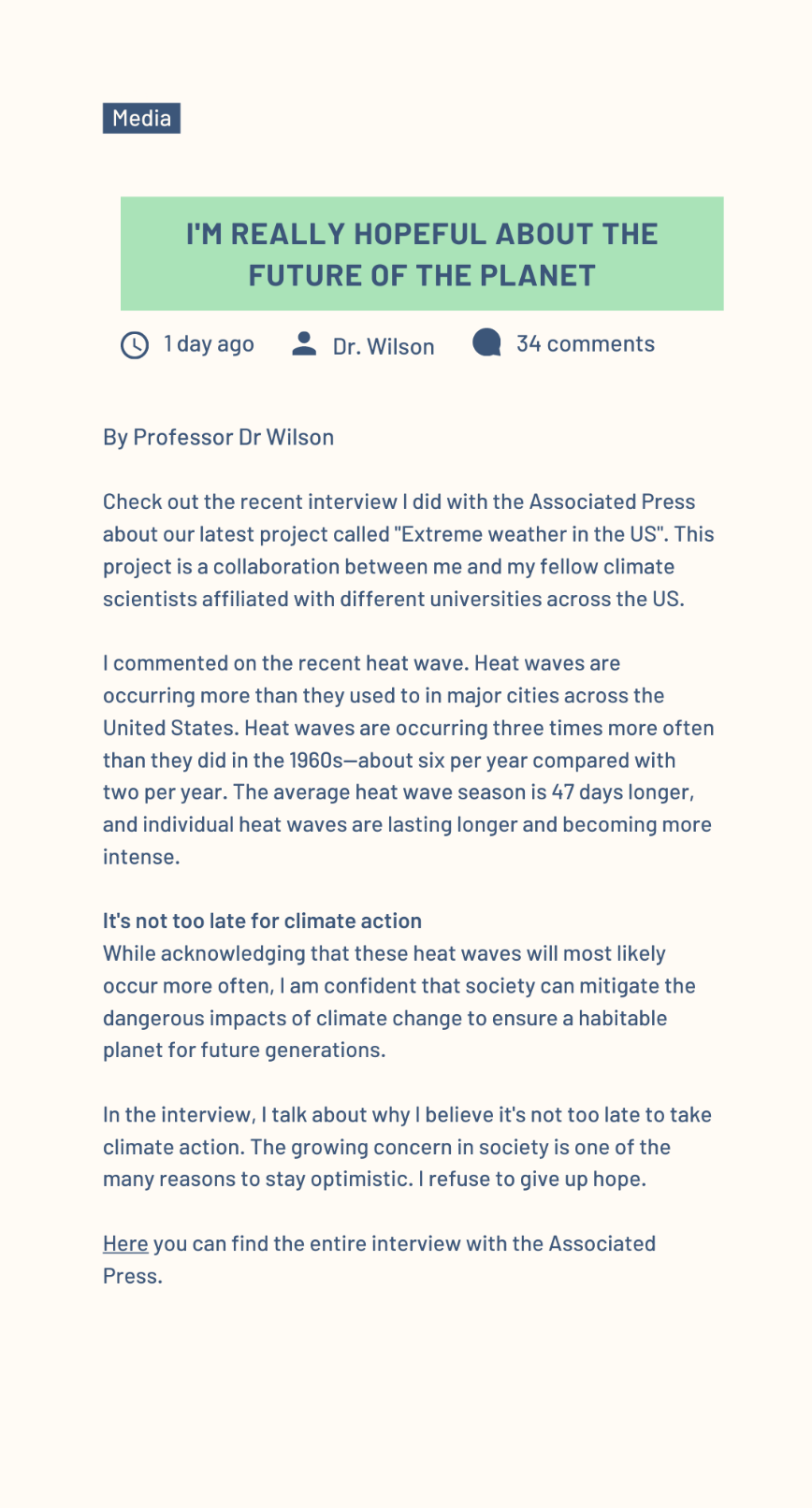


**Neutral x personal story**


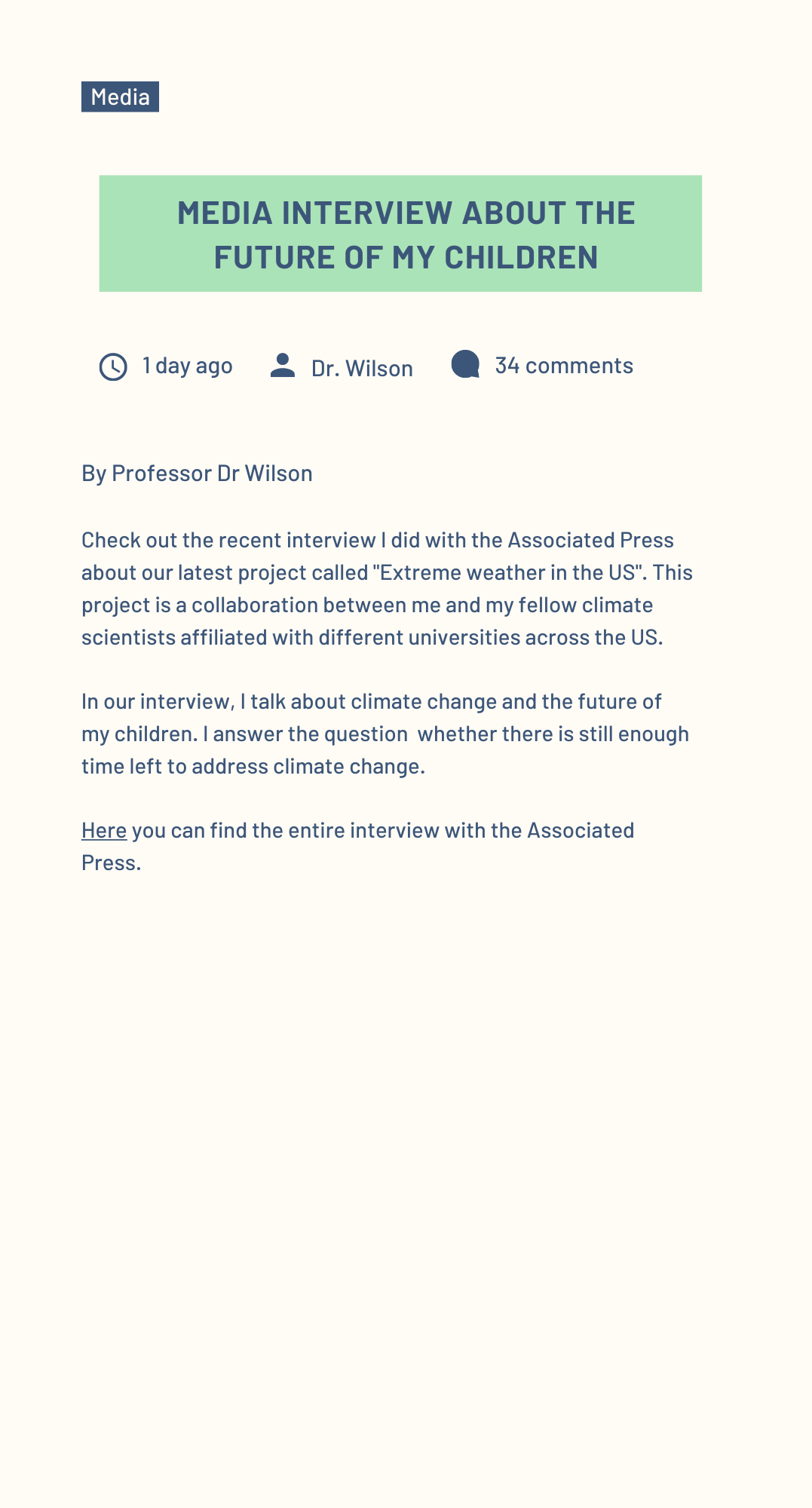


**Neutral x factual account**


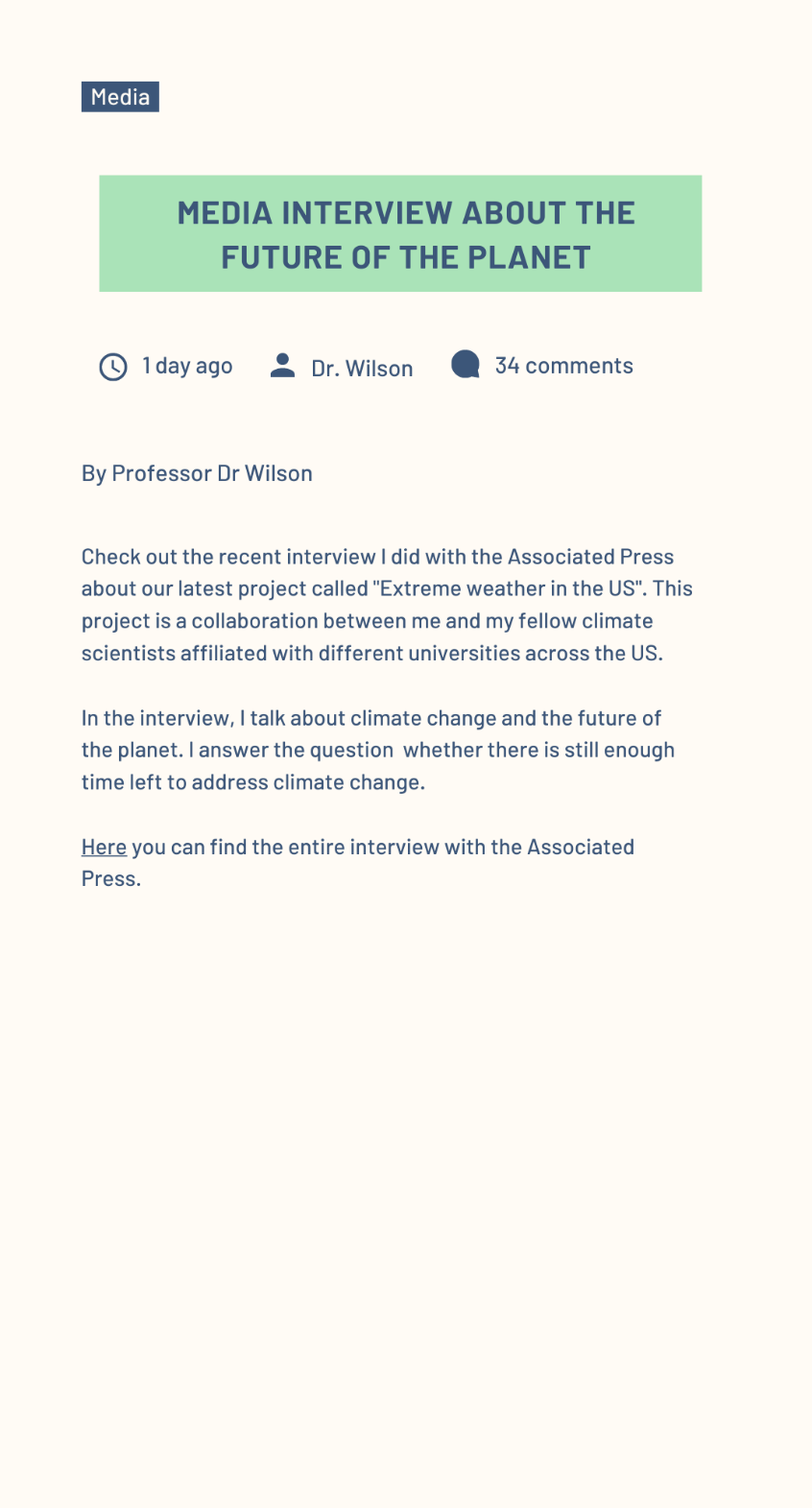


**Soft quotas and final sample U.S. general public**

|  | **Quota** | **%** | **Final sample** |
| --- | --- | --- | --- |
| **Age** |  |  |  |
| 18-24 | 102 | 12% | 11.2% |
| 25-34 | 153 | 18% | 19.8% |
| 35-44 | 138 | 16% | 17.3% |
| 45-54 | 141 | 17% | 16.7% |
| 55-64 | 143 | 17% | 15.5% |
| 65-99 | 172 | 20% | 18.7% |
| **Gender** |  |  |  |
| Male | 415 | 49% | 46.72% |
| Female | 435 | 51% | 46.8% |
| **Education level** |  |  |  |
| Low | 459 | 54% | 40.1% |
| Medium | 51 | 6% | 16.7% |
| High | 340 | 40% | 43.2% |
| **Total** | 850 | 100% |  |

**Manipulation checks**

The stimuli were pilot tested twice on MTurk with a smaller sample, to verify whether the stimuli were manipulated successfully. The first round of testing showed that the emotional expressions were not successfully manipulated. We initially focused on four distinct emotions (i.e., fear, hope, sadness, neutral) and had eight experimental conditions. However, respondents did not clearly register these distinct emotions. Therefore, instead of investigating the distinct emotions, we decided to focus on optimistic, pessimistic, and neutral affect (i.e., six experimental conditions) and changed the wording accordingly. The affective and narrative conditions were successfully manipulated in the second round of testing. We also ran the manipulation checks for the final dataset.

First, we verified whether respondents who were exposed to the affective conditions were indeed also more likely to indicate that Professor Wilson was pessimistic (*F*(2, 879) = [81.718], *p* = <0.001). A post-hoc comparison using a Bonferroni test indicated that the mean score for the pessimistic condition (*M* = 5.33, *SD* = 1.43) was significantly higher than the neutral condition (*M* = 4.39, *SD* = 1.37; *M_diff_ =* 0.94, *p* < 0.001) and optimistic condition (*M* = 3.72, *SD* = 1.8; *M_diff_ =* 0.67, *p* < 0.001).

The same analysis was conducted to see whether respondents who were exposed to the affective expression conditions were indeed also more likely to indicate that Professor Wilson was optimistic (*F*(2, 879) = [18.774], *p* = <0.001). A post-hoc comparison using a Bonferroni test indicated that the mean score for the optimistic condition (*M* = 5, *SD* = 1.58) was significantly higher than the neutral condition (*M* = 4.2, *SD* = 1.44; *M_diff_ =* 0.79, *p* < 0.001) and pessimistic condition (*M* = 3.41, *SD* = 1.44; *M_diff_ =* 1.59, *p* < 0.001).

Likewise, we investigated whether respondents who were exposed to the emotional expression conditions were indeed also more likely to indicate that Professor Wilson was emotional (*F*(2, 879) = [18.774], *p* = <0.001). A post-hoc comparison using a Bonferroni test indicated that the mean score for the neutral condition (*M* = 4.33, *SD* = 1.49) was significantly lower than the optimistic condition (*M* = 4.68, *SD* = 1.49; *M_diff_ =* -0.35, *p* = 0.012) and pessimistic condition (*M* = 5.08, *SD* = 1.45; *M_diff_ =* -0.75, *p* < 0.001).

There was a significant effect for all check questions at the p<.0.5 level, meaning that the emotional expression manipulation was successful.

Second, with two questions we verified whether respondents who were exposed to the personal story narrative were indeed also more likely to indicate that Professor Wilson explicitly talked about his own children (personal story *M =* 5.05; factual account *M =* 3.98; *F*(1, 880) = [90.374], *p* = <0.001);

and whether he shared his personal experiences with extreme weather events (personal story *M =* 4.89; factual account *M =* 4.27; *F*(1, 880) = [34.567], *p* = <0.001).

There was a significant effect for all check questions at the *p*<.05 level, meaning that the narrative form manipulation was successful.

**Tables**

|  | | | | |
| --- | --- | --- | --- | --- |
|  | *Dependent variable:* | | | |
|  |  | | | |
|  | Perceived credibility | Emotional arousal | Risk perception | Behavioral willingness |
|  | (1) | (2) | (3) | (4) |
|  | | | | |
| Neutral & Story^a^ | 0.09 | 0.01 | -0.23 | 0.60 |
|  | (0.34) | (0.48) | (0.42) | (0.44) |
|  |  |  |  |  |
| Optimistic & Fact^a^ | 0.02 | -0.36 | -0.32 | -0.17 |
|  | (0.33) | (0.47) | (0.41) | (0.43) |
|  |  |  |  |  |
| Optimistic & Story^a^ | 0.40 | 0.69 | 0.23 | 0.48 |
|  | (0.33) | (0.47) | (0.42) | (0.44) |
|  |  |  |  |  |
| Pessimistic & Fact^a^ | 0.30 | 0.77 | -0.08 | -0.24 |
|  | (0.34) | (0.48) | (0.43) | (0.45) |
|  |  |  |  |  |
| Pessimistic & Story^a^ | 0.02 | 0.19 | -0.48 | -0.04 |
|  | (0.35) | (0.50) | (0.44) | (0.46) |
|  |  |  |  |  |
| Personal experience | 0.18^**^ | 0.06 | 0.23^***^ | 0.17^*^ |
|  | (0.07) | (0.10) | (0.09) | (0.09) |
|  |  |  |  |  |
| Neutral & Story * Personal experience | -0.13 | -0.04 | -0.01 | -0.26^**^ |
|  | (0.09) | (0.13) | (0.12) | (0.12) |
|  |  |  |  |  |
| Optimistic & Fact * Personal experience | -0.06 | 0.05 | 0.01 | -0.09 |
|  | (0.09) | (0.13) | (0.11) | (0.12) |
|  |  |  |  |  |
| Optimistic & Story * Personal experience | -0.07 | -0.12 | -0.04 | -0.15 |
|  | (0.09) | (0.13) | (0.12) | (0.12) |
|  |  |  |  |  |
| Pessimistic & Fact * Personal experience | -0.10 | -0.05 | 0.004 | -0.001 |
|  | (0.09) | (0.13) | (0.12) | (0.12) |
|  |  |  |  |  |
| Pessimistic & Story * Personal experience | -0.01 | 0.13 | 0.13 | -0.01 |
|  | (0.10) | (0.14) | (0.12) | (0.13) |
|  |  |  |  |  |
| Constant | 4.46^****^ | 3.74^****^ | 4.14^****^ | 3.69^****^ |
|  | (0.25) | (0.35) | (0.31) | (0.32) |
|  |  |  |  |  |
|  | | | | |
| Observations | 882 | 882 | 882 | 882 |
| R^2^ | 0.04 | 0.04 | 0.07 | 0.03 |
| Adjusted R^2^ | 0.03 | 0.03 | 0.06 | 0.01 |
| Residual Std. Error (df = 870) | 1.21 | 1.72 | 1.52 | 1.59 |
| F Statistic (df = 11; 870) | 3.17^****^ | 3.60^****^ | 6.09^****^ | 2.04^**^ |
|  | | | | |
| ^a^Reference group is the neutral & factual condition | ^*^p<0.1; ^**^p<0.05; ^***^p<0.01 | | | |

**Table 1. Regression models for testing the moderating role of personal experiences with extreme weather events**

|  | | | | |
| --- | --- | --- | --- | --- |
|  | *Dependent variable:* | | | |
|  |  | | | |
|  | Perceived credibility | Emotional arousal | Risk perception | Behavioral willingness |
|  | (1) | (2) | (3) | (4) |
|  | | | | |
| Neutral & Story^a^ | -0.74^***^ | -0.52 | -0.21 | -0.03 |
|  | (0.27) | (0.40) | (0.32) | (0.33) |
|  |  |  |  |  |
| Optimistic & Fact^a^ | -0.27 | -0.24 | 0.19 | -0.73^**^ |
|  | (0.26) | (0.39) | (0.31) | (0.32) |
|  |  |  |  |  |
| Optimistic & Story^a^ | -0.27 | 0.03 | -0.15 | -0.48 |
|  | (0.26) | (0.38) | (0.31) | (0.32) |
|  |  |  |  |  |
| Pessimistic & Fact^a^ | 0.05 | 0.71^*^ | 0.37 | -0.27 |
|  | (0.27) | (0.39) | (0.31) | (0.33) |
|  |  |  |  |  |
| Pessimistic & Story^a^ | -0.19 | 0.76^*^ | 0.45 | -0.02 |
|  | (0.27) | (0.39) | (0.31) | (0.33) |
|  |  |  |  |  |
| Trust | -0.38^****^ | -0.35^****^ | -0.49^****^ | -0.57^****^ |
|  | (0.06) | (0.09) | (0.07) | (0.07) |
|  |  |  |  |  |
| Neutral & Story * Trust | 0.17^**^ | 0.17 | 0.04 | -0.01 |
|  | (0.08) | (0.12) | (0.10) | (0.10) |
|  |  |  |  |  |
| Optimistic & Fact * Trust | 0.06 | 0.04 | -0.12 | 0.14 |
|  | (0.08) | (0.12) | (0.09) | (0.10) |
|  |  |  |  |  |
| Optimistic & Story * Trust | 0.16^*^ | 0.10 | 0.09 | 0.18^*^ |
|  | (0.08) | (0.12) | (0.10) | (0.10) |
|  |  |  |  |  |
| Pessimistic & Fact * Trust | 0.01 | 0.001 | -0.09 | 0.07 |
|  | (0.08) | (0.12) | (0.09) | (0.10) |
|  |  |  |  |  |
| Pessimistic & Story * Trust | 0.07 | -0.04 | -0.16^*^ | 0.0001 |
|  | (0.08) | (0.12) | (0.10) | (0.10) |
|  |  |  |  |  |
| Constant | 6.06^****^ | 4.88^****^ | 6.23^****^ | 5.79^****^ |
|  | (0.18) | (0.27) | (0.22) | (0.23) |
|  |  |  |  |  |
|  | | | | |
| Observations | 882 | 882 | 882 | 882 |
| R^2^ | 0.18 | 0.12 | 0.31 | 0.27 |
| Adjusted R^2^ | 0.17 | 0.11 | 0.30 | 0.26 |
| Residual Std. Error (df = 870) | 1.12 | 1.65 | 1.31 | 1.37 |
| F Statistic (df = 11; 870) | 16.96^****^ | 10.69^****^ | 35.70^****^ | 29.68^****^ |
|  | | | | |
| ^a^Reference group is the neutral & factual condition | ^*^p<0.1; ^**^p<0.05; ^***^p<0.01 | | | |

**Table 2. Regression models for testing the moderating role of trust in the climate scientific community**

|  | | | | |
| --- | --- | --- | --- | --- |
|  | *Dependent variable:* | | | |
|  |  | | | |
|  | Perceived credibility | Emotional arousal | Risk perception | Behavioral willingness |
|  | (1) | (2) | (3) | (4) |
|  | | | | |
| Neutral & Story^a^ | 0.85 | 0.19 | 0.98 | -0.21 |
|  | (1.55) | (2.17) | (1.98) | (2.02) |
|  |  |  |  |  |
| Optimistic & Fact^a^ | 1.41 | -1.92 | -0.05 | 0.88 |
|  | (1.51) | (2.11) | (1.93) | (1.97) |
|  |  |  |  |  |
| Optimistic & Story^a^ | 2.27 | 1.14 | 2.96 | 2.78 |
|  | (1.46) | (2.05) | (1.87) | (1.91) |
|  |  |  |  |  |
| Pessimistic & Fact^a^ | 0.50 | 1.75 | 1.39 | -0.43 |
|  | (1.53) | (2.15) | (1.97) | (2.01) |
|  |  |  |  |  |
| Pessimistic & Story^a^ | 0.41 | -0.09 | 2.10 | -0.82 |
|  | (1.49) | (2.08) | (1.91) | (1.95) |
|  |  |  |  |  |
| Optimistic trait | 0.19^***^ | 0.20^**^ | 0.24^***^ | 0.20^**^ |
|  | (0.07) | (0.10) | (0.09) | (0.09) |
|  |  |  |  |  |
| Neutral & Story * Optimistic trait | -0.08 | -0.02 | -0.08 | -0.003 |
|  | (0.10) | (0.14) | (0.12) | (0.13) |
|  |  |  |  |  |
| Optimistic & Fact * Optimistic trait | -0.10 | 0.11 | -0.01 | -0.08 |
|  | (0.10) | (0.13) | (0.12) | (0.12) |
|  |  |  |  |  |
| Optimistic & Story * Optimistic trait | -0.13 | -0.05 | -0.18 | -0.18 |
|  | (0.09) | (0.13) | (0.12) | (0.12) |
|  |  |  |  |  |
| Pessimistic & Fact * Optimistic trait | -0.03 | -0.07 | -0.09 | 0.01 |
|  | (0.10) | (0.13) | (0.12) | (0.13) |
|  |  |  |  |  |
| Pessimistic & Story * Optimistic trait | -0.03 | 0.04 | -0.14 | 0.04 |
|  | (0.09) | (0.13) | (0.12) | (0.12) |
|  |  |  |  |  |
| Constant | 2.05^*^ | 0.76 | 1.10 | 1.14 |
|  | (1.08) | (1.52) | (1.39) | (1.42) |
|  |  |  |  |  |
|  | | | | |
| Observations | 882 | 882 | 882 | 882 |
| R^2^ | 0.04 | 0.07 | 0.03 | 0.04 |
| Adjusted R^2^ | 0.03 | 0.06 | 0.02 | 0.03 |
| Residual Std. Error (df = 870) | 1.21 | 1.69 | 1.55 | 1.58 |
| F Statistic (df = 11; 870) | 3.52^****^ | 5.97^****^ | 2.76^***^ | 3.15^****^ |
|  | | | | |
| ^a^Reference group is the neutral & factual condition | ^*^p<0.1; ^**^p<0.05; ^***^p<0.01 | | | |

**Table 3. Regression models for testing the moderating role of optimistic trait**
